# Supplementary material for: Genetic Characterization and Mating Disruption in Spodoptera Species, a Case Study on Spodoptera frugiperda (Lepidoptera, Noctuidae): A Systematic Review
Source: Insects. 2025 Nov 18;16(11):1176. doi: 10.3390/insects16111176 (PMC12653782; doi:10.3390/insects16111176)
Supplement: Supplementary file 1 [file insects-16-01176-s001.zip › insects-3899740-PRISMA 2020 Main Checklist.pdf]

PRISMA 2020 Main Checklist

Topic	No.	Item	Location where item is reported
**TITLE**			
**Title**	1	Identify the report as a systematic review.	Page 1, Line 3
**ABSTRACT**			
**Abstract**	2	See the PRISMA 2020 for Abstracts checklist	Page 1
**INTRODUCTION**			
**Rationale**	3	Describe the rationale for the review in the context of existing knowledge.	Section 1, Lines 45-118
**Objectives**	4	Provide an explicit statement of the objective(s) or question(s) the review addresses.	Section 1, Lines 119-130
**METHODS**			
**Eligibility criteria**	5	Specify the inclusion and exclusion criteria for the review and how studies were grouped for the syntheses.	Section 2.2, Lines 160-174
**Information sources**	6	Specify all databases, registers, websites, organizations, reference lists and other sources searched or consulted to identify studies. Specify the date when each source was last searched or consulted.	Section 2.1, Lines 138-141
**Search strategy**	7	Present the full search strategies for all databases, registers and websites, including any filters and limits used.	Section 2.1, Lines 133-152
**Selection process**	8	Specify the methods used to decide whether a study met the inclusion criteria of the review, including how many reviewers screened each record and each report retrieved, whether they worked independently, and if applicable, details of automation tools used in the process.	Section 2.2, Lines 159-152
**Data collection process**	9	Specify the methods used to collect data from reports, including how many reviewers collected data from each report, whether they worked independently, any processes for obtaining or confirming data from study investigators, and if applicable, details of automation tools used in the process.	Section 2.3, Lines 187-201

Topic	No.	Item	Location where item is reported
**Data items**	10a	List and define all outcomes for which data were sought. Specify whether all results that were compatible with each outcome domain in each study were sought (e.g. for all measures, time points, analyses), and if not, the methods used to decide which results to collect.	Section 3.1.1, Lines 193-208
	10b	List and define all other variables for which data were sought (e.g. participant and intervention characteristics, funding sources). Describe any assumptions made about any missing or unclear information.	Table S3 data extraction in supplementary data describes variables such as from genetic and pheromone studies
**Study risk of bias assessment**	11	Specify the methods used to assess risk of bias in the included studies, including details of the tool(s) used, how many reviewers assessed each study and whether they worked independently, and if applicable, details of automation tools used in the process.	Not explicitly discussed in the manuscript
**Effect measures**	12	Specify for each outcome the effect measure(s) (e.g. risk ratio, mean difference) used in the synthesis or presentation of results.	Not applicable. The study presents descriptive results and does not perform statistical synthesis or specify effect measures such as risk ratios or mean differences.
**Synthesis methods**	13a	Describe the processes used to decide which studies were eligible for each synthesis (e.g. tabulating the study intervention characteristics and comparing against the planned groups for each synthesis (item 5)).	Section 2.2, Lines 145-165.<br>Presents results through tables and diagrams; meta-analysis not performed
	13b	Describe any methods required to prepare the data for presentation or synthesis, such as handling of missing summary statistics, or data conversions.	Section 2.3, Lines 193-208<br>Data were prepared using tools like Excel
	13c	Describe any methods used to tabulate or visually display results of individual studies and syntheses.	Section 2.4, Lines 210-220.<br>Results were presented by Excel tables, graphics and diagrams, using various tools such Microsoft Excel (v. 2403), R software (v. 4.2.3), QGIS software v.3.28.4 and RAWGraphs
	13d	Describe any methods used to synthesize results and provide a rationale for the choice(s). If meta-analysis was performed, describe the model(s), method(s) to identify the presence and extent of statistical heterogeneity, and software package(s) used.	Section 3<br>Results were synthesized descriptively, emphasizing trends in main topics, without performing a meta-analysis due to the heterogeneity of study designs and outcomes
	13e	Describe any methods used to explore possible causes of heterogeneity among study results (e.g. subgroup analysis, meta-regression).	Section 3.2.1<br>Heterogeneity was explored by examining data variations, but detailed subgroup analyses or meta-regressions were not performed

Topic	No.	Item	Location where item is reported
	13f	Describe any sensitivity analyses conducted to assess robustness of the synthesized results.	Not applicable.<br>Sensitivity analyses were not performed as the review did not include meta-analytic statistical syntheses
**Reporting bias assessment**	14	Describe any methods used to assess risk of bias due to missing results in a synthesis (arising from reporting biases).	Not explicitly assessed in the manuscript
**Certainty assessment**	15	Describe any methods used to assess certainty (or confidence) in the body of evidence for an outcome.	Not explicitly assessed in the manuscript
**RESULTS**			
**Study selection**	16a	Describe the results of the search and selection process, from the number of records identified in the search to the number of studies included in the review, ideally using a flow diagram.	Section 3.1, Lines 224-247.<br>Includes a PRISMA flow diagram Section 2.2 Line185
	16b	Cite studies that might appear to meet the inclusion criteria, but which were excluded, and explain why they were excluded.	Section 2.2, Line 163-174
**Study characteristics**	17	Cite each included study and present its characteristics.	Section 3.1.1 - Lines 223-293
**Risk of bias in studies**	18	Present assessments of risk of bias for each included study.	Not discussed in the manuscript
**Results of individual studies**	19	For all outcomes, present, for each study: (a) summary statistics for each group (where appropriate) and (b) an effect estimates and its precision (e.g. confidence/credible interval), ideally using structured tables or plots.	Section 3.1.2.<br>Details presented in tables and figures
**Results of syntheses**	20a	For each synthesis, briefly summarize the characteristics and risk of bias among contributing studies.	Section 3.2.2<br>Summarizes the characteristics of studies on genetic markers and pheromone components but does not explicitly address bias. Combined results presented descriptively and visually
	20b	Present results of all statistical syntheses conducted. If meta-analysis was done, present for each the summary estimate and its precision (e.g. confidence/credible interval) and measures of statistical heterogeneity. If comparing groups, describe the direction of the effect.	Not applicable.<br>Meta-analysis was not performed, and statistical results were not synthesized
	20c	Present results of all investigations of possible causes of heterogeneity among study results.	Section 3.2.1<br>Limited discussion on heterogeneity
	20d	Present results of all sensitivity analyses conducted to assess the robustness of the synthesized results.	Not applicable.<br>Sensitivity analyses were not conducted as meta-analysis was not performed

Topic	No.	Item	Location where item is reported
**Reporting biases**	21	Present assessments of risk of bias due to missing results (arising from reporting biases) for each synthesis assessed.	Not discussed in the manuscript
**Certainty of evidence**	22	Present assessments of certainty (or confidence) in the body of evidence for each outcome assessed.	Not discussed in the manuscript
**DISCUSSION**			
**Discussion**	23a	Provide a general interpretation of the results in the context of other evidence.	Section 3 (3.21; 3.2.2, 3.2.3 and 3.2.4)<br>Interprets findings in the context of existing literature.
	23b	Discuss any limitations of the evidence included in the review.	Section 4. Discusses gaps in research
	23c	Discuss any limitations of the review processes used.	Section 4. Addresses limitations in review methodology
	23d	Discuss implications of the results for practice, policy, and future research.	Section 4.
**OTHER INFORMATION**			
**Registration and protocol**	24a	Provide registration information for the review, including register name and registration number, or state that the review was not registered.	Not registered;
	24b	Indicate where the review protocol can be accessed, or state that a protocol was not prepared.	Protocol details provided online repository Figshare Dataset. https://doi.org/10.6084/m9.figshare.28135550.v1
	24c	Describe and explain any amendments to information provided at registration or in the protocol.	
**Support**	25	Describe sources of financial or non-financial support for the review, and the role of the funders or sponsors in the review.	“Funding” section, Lines 652-663
**Competing interests**	26	Declare any competing interests of review authors.	“Declaration of Competing Interests” section, Line 669-670
**Availability of data, code and other materials**	27	Report which of the following are publicly available and where they can be found: template data collection forms; data extracted from included studies; data used for all analyses; analytic code; any other materials used in the review.	“Data Availability” section, Line 664-665.<br>Supplementary materials and data are available online repository Figshare Dataset. https://doi.org/10.6084/m9.figshare.28135550.v1

PRIMSA Abstract Checklist

Topic	No.	Item	Reported?
**TITLE**			
**Title**	1	Identify the report as a systematic review.	Yes
**BACKGROUND**			
**Objectives**	2	Provide an explicit statement of the main objective(s) or question(s) the review addresses.	Yes
**METHODS**			
**Eligibility criteria**	3	Specify the inclusion and exclusion criteria for the review.	Yes
**Information sources**	4	Specify the information sources (e.g. databases, registers) used to identify studies and the date when each was last searched.	Yes
**Risk of bias**	5	Specify the methods used to assess risk of bias in the included studies.	No
**Synthesis of results**	6	Specify the methods used to present and synthesize results.	Yes
**RESULTS**			
**Included studies**	7	Give the total number of included studies and participants and summarise relevant characteristics of studies.	Yes
**Synthesis of results**	8	Present results for main outcomes, preferably indicating the number of included studies and participants for each. If meta-analysis was done, report the summary estimate and confidence/credible interval. If comparing groups, indicate the direction of the effect (i.e. which group is favoured).	Yes
**DISCUSSION**			
**Limitations of evidence**	9	Provide a brief summary of the limitations of the evidence included in the review (e.g. study risk of bias, inconsistency and imprecision).	Yes
**Interpretation**	10	Provide a general interpretation of the results and important implications.	Yes
**OTHER**			
**Funding**	11	Specify the primary source of funding for the review.	Yes
**Registration**	12	Provide the register name and registration number.	No

From: Page MJ, McKenzie JE, Bossuyt PM, Boutron I, Hoffmann TC, Mulrow CD, et al. The PRISMA 2020 statement: an updated guideline for reporting systematic reviews. MetaArXiv. 2020, September 14. DOI: 10.31222/osf.io/v7gm2. For more information, visit: www.prisma-statement.org
