# Supplementary material for: Genetic Characterization and Mating Disruption in Spodoptera Species, a Case Study on Spodoptera frugiperda (Lepidoptera, Noctuidae): A Systematic Review
Source: Insects. 2025 Nov 18;16(11):1176. doi: 10.3390/insects16111176 (PMC12653782; doi:10.3390/insects16111176)
Supplement: Supplementary file 1 [file insects-16-01176-s001.zip › Supplementary material.pdf]

Supplementary material

Tables

Table S1. Number of publications retrieved from the online databases using the specific keywords and the respective Boolean operators.

Categories	Keywords	Scopus	N. of articles	Web of Science	N. of articles	CABI Digital Library	N. of articles
1) Genetic characterization and strain identification in Spodoptera species	("Spodoptera" OR "Spodoptera frugiperda" OR "fall armyworm") AND ("genetic characterization" OR "molecular identification" OR "strain identification" OR "genetic marker")	(("Spodoptera" OR "Spodoptera frugiperda" OR "fall armyworm") AND ("genetic characterization" OR "molecular identification" OR "strain identification" OR "genetic marker")) AND (LIMIT-TO (DOCTYPE, "ar") OR LIMIT-TO (DOCTYPE , "re"))	2,547	(("Spodoptera" OR "Spodoptera frugiperda" OR "fall armyworm") AND ("genetic characterization" OR "molecular identification" OR "strain identification" OR "genetic marker"))	103	[[All: "spodoptera"] OR [All: "spodoptera frugiperda"] OR [All: "fall armyworm"]] AND [[All: "genetic characterization"] OR [All: "molecular identification"] OR [All: "strain identification"] OR [All: "genetic marker"]] AND [CABI Products: CAB Abstracts] AND [Item Type: Journal Article]	69
2) Sex pheromone and manting disruption in Spodoptera species	("Spodoptera" OR "Spodoptera frugiperda" OR "fall armyworm") AND ("Sex pheromone" AND "mating disruption")	("Spodoptera" OR "Spodoptera frugiperda" OR "fall armyworm") AND ("Sex pheromone" AND "mating disruption") AND (LIMIT-TO (DOCTYPE, "ar") OR LIMIT-TO (DOCTYPE, "re"))	525	(("Spodoptera" OR "Spodoptera frugiperda" OR "fall armyworm") AND ("Sex pheromone" AND "mating disruption"))	538	[[All: "spodoptera"] OR [All: "spodoptera frugiperda"] OR [All: "fall armyworm"]] AND [All: "sex pheromone"] AND [All: "mating disruption"] AND [CABI Products: CAB Abstracts] AND [Item Type: Journal Article]	456
3) The effectiveness of the sex pheromone for mating disruption according to the genetic characterization and strain identified in Spodoptera species	("Spodoptera" OR "Spodoptera frugiperda" OR "fall armyworm") AND ("genetic characterization" OR "strain identification") AND ("Sex pheromone" OR "mating disruption")	("Spodoptera" OR "Spodoptera frugiperda" OR "fall armyworm") AND ("genetic characterization" OR "strain identification") AND ("Sex pheromone" OR "mating disruption") AND (LIMIT-TO (DOCTYPE, "ar") OR LIMIT-TO (DOCTYPE , "re"))	183	(("Spodoptera" OR "Spodoptera frugiperda" OR "fall armyworm") AND ("genetic characterization" OR "strain identification") AND ("Sex pheromone" OR "mating disruption"))	46	[[All: "spodoptera"] OR [All: "spodoptera frugiperda"] OR [All: "fall armyworm"]] AND [[All: "genetic characterization"] OR [[All: "strain identification"] AND [All: "sex pheromone"]] OR [All: "mating disruption"]] AND [CABI Products: CAB Abstracts] AND [Item Type: Journal Article]	56
		**Total articles on Scopus**	**3,255**	**Total articles on WoS**	**687**	**Total articles on CABI**	**581**
	**Total articles identified**	**4.523**					

Table S2. The list of articles included in this review list of articles included in the review, indicating the topic of the related study, with their respective references (authors, year of publication, title, publishing source and DOI access link)

ID	Study topic	Author(s)	Year of Publication	Title	Source/Journal	DOI/ Access link
1	B (Phe)	Kawasaki, K., Miyashita, K.	1976	Mating suppression by individual components of sex-pheromone of *Spodoptera litura* f (Lepidoptera-Noctuidae) under field conditions	Applied Entomology and Zoology	https://doi.org/10.1303/aez.11.320
2	B (Phe)	Nakamura,. K	1976	The effect of wind velocity on diffusion of *Spodoptera litura* (f) sex-pheromone	Applied Entomology and Zoology	https://doi.org/10.1303/aez.11.312
3	B (Phe)	Kawasaki, K.	1981	A functional difference of the individual components of *Spodoptera litura* (f) (Lepidoptera, Noctuidae) sex-pheromone in the attraction of flying male moths	Applied Entomology and Zoology	https://doi.org/10.1303/aez.16.63
4	B (Phe)	Tumlinson, J.H., Mitchell, E.R., Teal, P.E..A, Heath, R.R., Mengelkoch, L.J.	1986	Sex pheromone of fall armyworm, *Spodoptera frugiperda* (J.E. Smith): Identification of components critical to attraction in the field	Journal of Chemical Ecology	https://doi.org/10.1007/BF01041855
5	B (Phe)	Wakamura, S., Kozai, S., Inoue, H., Takai, M., Yamashita, I., Kawahara, S., Kawamur. M.	1989	Control of the Beet Armyworm, *Spodoptera exigua* (HUEBNER) (Lepidoptera: Noctuidae), Using Synthetic Sex Pheromone. I. Effect of Communication Disruption in Welsh Onion Fields	Applied Entomology and Zoology	https://doi.org/10.1303/aez.24.387
6	B (Phe)	Wakamura, S., Takai. M.	1995	Communication disruption for control of the beet armyworm, *Spodoptera exigua* (hubner), with synthetic sex-pheromone	Jarq-Japan Agricultural Research Quarterly	ISSN 1721-8861 https://www.jircas.go.jp/sites/default/files/publication/jarq/29-2-125-130_0.pdf
7	A (Gen)	Yanghang, L., Adang, M.J.	1996	Distinguishing Fall Armyworm (Lepidoptera: Noctuidae) Strains Using a Diagnostic Mitochondrial DNA Marker	Florida Entomologist	https://doi.org/10.2307/3495753
8	B (Phe)	Mitchell, R.E., Mayer, S.M.	2001	*Spodoptera exigua*: mating disruption, measurement of airborne concentration of pheromone, and use of specialist receptor cell responses for comparison to female pheromone emission	Journal of Environmental Science and Health	https://doi.org/10.1081/PFC-100104190
9	B (Phe)	Anderson, P., Sadek, M.M., Hansson, B.S.	2003	Pre-exposure modulates attraction to sex pheromone in a moth	Chemical Senses	https://doi.org/10.1093/chemse/28.4.285
10	A (Gen)	Levy, H.C., Garcia-Maruniak, A., Maruniak, J.E.	2003	Strain identification of *Spodoptera frugiperda* (Lepidoptera: Noctuidae) insects and cell line: PCR-RFLP of cytochrome oxidase C subunit I gene	Florida Entomologist	https://doi.org/10.1653/0015-4040(2002)085[0186:siosfl]2.0.co;2

11	A (Gen)	Meagher, Jr. R.L., Gallo-Meagher, M.	2003	Identifying host strains of fall armyworm (Lepidoptera: Noctuidae) in Florida using mitochondrial markers	Florida Entomologist	https://doi.org/10.1653/0015-4040(2003)086[0450:IHSOFA]2.0.CO;2
12	A (Gen)	Busato, G.R., Grützmacher, A.D., De Oliveira, A.C., Vieira, E.A., Zimmer, P.D., Kopp, M.M., Bandeira, J.M., Magalhães, T.R.	2004	Analysis of the molecular structure and diversity of *Spodoptera frugiperda* (J.E. Smith) (Lepidoptera: Noctuidae) populations associated to the corn and rice crops in Rio Grande do Sul state, Brazil; [Análise da estrutura e diversidade molecular de populações de *Spodoptera frugiperda* (J.E. Smith) (Lepidoptera: Noctuidae) associadas às culturas de milho e arroz no Rio Grande do Sul]	Neotropical Entomology	https://doi.org/10.1590/S1519-566X2004000600008
13	A (Gen)	Martineli, S., Silva-Filho, M.C.	2006	Molecular Variability of *Spodoptera frugiperda* (Lepidoptera: Noctuidae) Populations Associated to Maize and Cotton Crops in Brazil	Molecular Entomology	https://doi.org/10.1603/0022-0493-99.2.519
14	A (Gen)	Lewter, J.A., Szalanski, A.L.	2007	Molecular identification of the fall armyworm, *Spodoptera frugiperda* (J.E. Smith) (Lepidoptera: Noctuidae) using PCR-RFLP	Journal of Agricultural and Urban Entomology	https://doi.org/10.3954/1523-5475-24.2.51
15	A (Gen)	Nagoshi, R.N., Silvie, P., Meagher, R.L., Lopez, J., Machado, V.	2007	Identification and comparison of fall armyworm (Lepidoptera: Noctuidae) host strains in Brazil, Texas, and Florida	Annals of the Entomological Society of America	https://doi.org/10.1603/0013-8746(2007)100[394:IACOFA]2.0.CO;2
16	A (Gen)	Machado, V., Wunder, M., Baldissera, V.D., Oliveira, J.V., Fiúza, L.M., Nagoshi, R.N.	2008	Molecular characterization of host strains of *Spodoptera frugiperda* (Lepidoptera: Noctuidae) in southern Brazil	Annals of the Entomological Society of America	https://doi.org/10.1603/0013-8746(2008)101[619:MCOHSO]2.0.CO;2
17	A (Gen)	Vélez-Arango, A.M., Arango, R.E., Villanueva, D.M., Aguilera, E.G., Saldamando, C.I.B.	2008	Identification of *Spodoptera frugiperda* biotypes (Lepidoptera: Noctuidae) through using mitochondrial and nuclear markers	Revista Colombiana de Entomología	https://www.researchgate.net/publication/257652277
18	A (Gen)	Nagoshi, R.N.	2010	The fall armyworm triose phosphate isomerase (Tpi) gene as a marker of strain identity and interstrain mating	Annals of the Entomological Society of America	https://doi.org/10.1603/AN09046
19	A (Gen)	Saldamando, C.I., Vélez-Arango, A.M.	2010	Host plant association and genetic differentiation of corn and rice strains of *Spodoptera frugiperda* Smith (Lepidoptera: Noctuidae) in Colombia	Neotropical Entomology	https://doi.org/10.1590/S1519-566X2010000600012
20	A (Gen)	Arias, R.S., Blanco, C.A., Portilla, M., Snodgrass, G.L., Scheffler, B.E.	2011	First microsatellites from *Spodoptera frugiperda* (Lepidoptera: Noctuidae) and their potential use for population genetics	Annals of the Entomological Society of America	https://doi.org/10.1603/AN10135
21	A (Gen)	Nagoshi, R.N., Brambila, J., Meagher, R.L.	2011	Use of DNA barcodes to identify invasive armyworm *Spodoptera* species in Florida	Journal of Insect Science	https://doi.org/10.1673/031.011.15401

22	A (Gen)	Salinas-Hernandez, H., Saldamando-Benjumea, C.I.	2011	Haplotype identification within *Spodoptera frugiperda* (j.e. smith) (Lepidoptera: Noctuidae) corn and rice strains from Colombia	Neotropical Entomology	https://doi.org/10.1590/S1519-566X2011000400002
23	A (Gen)	Belay, D.K., Clark, P.L., Skoda, S.R., Isenhour, D.J., Molina-Ochoa, J., Gianni, C., Foster, J.E.	2012	Spatial genetic variation among *Spodoptera frugiperda* (Lepidoptera: Noctuidae) sampled from the United States, Puerto Rico, Panama, and Argentina	Annals of the Entomological Society of America	https://doi.org/10.1603/AN11111
24	A (Gen)	Juarez, L.M., Murua, G.M., Garcia, M.G., Ontivero, M., Vera, M.T., Vilardi, J.C., Groot, A.T., Castagnaro, A.P., Gastaminza, G., Willink, E.	2012	Host Association of *Spodoptera frugiperda* (Lepidoptera: Noctuidae) Corn and Rice Strains in Argentina, Brazil, and Paraguay	Journal of Economic Entomology	https://doi.org/10.1603/EC11184
25	B (Phe)	Lanzoni, A., Bazzocchi, G.G., Reggiori, F., Rama, F., Sannino, L., Maini, S., Burgio, G.	2012	*Spodoptera littoralis* male capture suppression in processing spinach using two kinds of synthetic sex-pheromone dispensers	Bulletin of Insectology	https://www.academia.edu/8268269 5/ ISSN 1721-8861
26	A (Gen)	Lobo-Hernández, M.I., Benjumea, C.I.S.	2012	Molecular Characterization and Genetic Differentiation of *Spodoptera frugiperda* J. E. Smith (Lepidoptera: Noctuidae) in Maize, Rice, and Cotton Fields of Colombia with AFLP	Southwestern Entomologist	https://doi.org/10.3958/059.037.021 3
27	A (Gen)	Nagoshi, R.N., Murúa, M. G., Hay-Roe, M., Juárez, M. L., Willink, E., Meagher, R.L.	2012	Genetic characterization of fall armyworm (Lepidoptera: Noctuidae) host strains in Argentina	Journal of Economic Entomology	https://doi.org/10.1603/EC11332
28	A (Gen)	Loto, F., Romero, C.M., Baigorí, M.D., Pera, L.M.	2013	Direct DNA amplification from fall armyworm (Lepidoptera: Noctuidae) samples	Florida Entomologist	https://doi.org/10.1653/024.096.045 2
29	C (Gen + Phe)	Meagher, R.L., Nagoshi, R.N., Armstrong, J.S., Niogret, J., Epsky, N.D., Flanders, K.L.	2013	Captures and Host Strains of Fall Armyworm (Lepidoptera: Noctuidae) Males in Traps Baited with Different Commercial Pheromone Blends	Florida Entomologist	https://doi.org/10.1653/024.096.030 5
30	A (Gen)	Pavinato, V.A.C., Martinelli, S., De Lima, P.F., Zucchi, M.I., Omoto, C.	2013	Microsatellite markers for genetic studies of the fall armyworm, *Spodoptera frugiperda*	Genetics and Molecular Research	https://doi.org/10.4238/2013.Februa ry.8.1
31	C (Gen + Phe)	Unbehend, M., Hänniger, S., Meagher, R. L., Heckel, D.G., Groot, A.T.	2013	Pheromonal Divergence Between Two Strains of *Spodoptera frugiperda*	Journal of Chemichal Ecology	https://doi.org/10.1007/s10886-013-0263-6
32	A (Gen)	Nagoshi, R.N., Meagher, R.L., Hay-Roe, M.	2014	Assessing the resolution of haplotype distributions to delineate fall armyworm (Lepidoptera: Noctuidae) migratory behaviors	Journal of Economic Entomology	https://doi.org/10.1603/EC14124

33	C (Gen + Phe)	Saldamando-Benjumea, C.I., Estrada-Piedrahíta, K.,Velásquez-Vélez, M.I., Bailey, R.I.	2014	Assortative Mating and Lack of Temporality Between Corn and Rice Strains of *Spodoptera frugiperda* (Lepidoptera, Noctuidae) from Central Colombia	Journal of Insect Behavior	https://doi.org/10.1007/s10905-014-9451-7
34	A (Gen)	Van De Vossenberg, B.T.L.H., Van Der Straten, M.J.	2014	Development and validation of real-time PCR tests for the identification of four *Spodoptera* species: *Spodoptera eridania, Spodoptera frugiperda, Spodoptera littoralis*, and *Spodoptera litura* (Lepidoptera: Noctuidae)	Journal of Economic Entomology	https://doi.org/10.1603/EC14132
35	A (Gen)	Cano-Calle, D., Arango-Isaza, R.E., Saldamando-Benjumea, C.I.	2015	Molecular identification of *Spodoptera frugiperda* (Lepidoptera: Noctuidae) corn and rice strains in Colombia by using a PCR-RFLP of the mitochondrial gene cytochrome oxydase i (COI) and a PCR of the gene FR (For Rice)	Annals of the Entomological Society of America	https://doi.org/10.1093/aesa/sav001
36	A (Gen)	Dumas, P., Legeai, F., Lemaitre, C., Scaon, E., Orsucci, M., Labadie, K., Gimenez, S., Clamens, A.-L., Henri, H., Vavre, F., Aury, J.-M., Fournier, P., Kergoat, G.J., d'Alençon, E.	2015	*Spodoptera frugiperda* (Lepidoptera: Noctuidae) host-plant variants: two host strains or two distinct species?	Genetica	https://doi.org/10.1007/s10709-015-9829-2
37	A (Gen)	Dumas, P., Barbut, J., Le, R.B., Silvain, J.-F., Clamens, A.-L., d'Alençon, E., Kergoat, G.J.	2015	Phylogenetic Molecular Species Delimitations Unravel Potential New Species in the Pest Genus *Spodoptera* Guenée, 1852 (Lepidoptera, Noctuidae)	PLoS ONE	https://doi.org/10.1371/journal.pone.0122407
38	A (Gen)	Nagoshi, R.N., Rosas-García, N.M., Meagher, R.L., Fleischer, S.J., Westbrook, J.K., Sappington, T.W., Hay-Roe, M., Thomas, J.M.G., Murúa, G.M.	2015	Haplotype profile comparisons between *Spodoptera frugiperda* (Lepidoptera: Noctuidae) populations from Mexico with those from Puerto Rico, South America, and the United States and their implications to migratory behavior	Journal of Economic Entomology	https://doi.org/10.1093/jee/tou044
39	A (Gen)	Pecina-Quintero, V., Anaya-López, J.L., Cortez-Mondaca, E., Nuñez-Colín, A., Herrera-Corredor, C., Montes-García, N., Jimenez-Becerril, M.F., Gámez-Vazquez, A.J.	2015	Caracterización Genética de *Spodoptera frugiperda* (Lepidoptera: Noctuidae) en México Usando Marcadores AFLP	Southwestern Entomologist	https://doi.org/10.3958/059.040.0313
40	A (Gen)	Shashank, P.R., Thomas, A., Ramamurthy, V.V.	2015	DNA barcoding and phylogenetic relationships of *Spodoptera litura* and *S. exigua* (Lepidoptera: Noctuidae)	Florida Entomologist	https://doi.org/10.1653/024.098.0138
41	A (Gen)	Bapatla, K.G., Patil, R.H.	2016	Genetic diversity in *Spodoptera litura* (Fab.) from major soybean growing states of India	Legume Research	https://doi.org/10.18805/lr.v0i0.7651

42	B (Phe)	Blassioli-Moraes, M.C., Barges, M., Viana, A.R., Laumann, R.A., Miranda, J.E., Magalhaes, D.M., Birkett, M.A.	2016	Identification and field evaluation of the sex pheromone of a Brazilian population of *Spodoptera cosmioides*	Pesquisa Agropecuaria Brasileira	https://doi.org/10.1590/S0100-204X2016000500015
43	A (Gen)	Perez-Zubiri, J.R., Cerna-Chavez, E., Aguirre-Uribe, L.A., Landeros-Flores, J., Harris, M.K., Rodriguez-Herrera, R.	2016	Population Variability of *Spodoptera frugiperda* (Lepidoptera: Noctuidae) in Maize (Poales: Poaceae) Associated with the Use of Chemical Insecticides	Florida Entomologist	https://doi.org/10.1653/024.099.0233
44	A (Gen)	Queiroz, P.R., Ramiro, C.A., Martins, E.S., Soberón, M., Bravo, A., Monnerat, R.G.,	2016	Mitochondrial markers to distinguish *Spodoptera frugiperda* populations associated with corn and cotton crops	Pesquisa Agropecuaria Brasileira	https://doi.org/10.1590/S0100-204X2016000500035
45	A (Gen)	Zhou, L.-H., Wang, X.-Y., Lei, J.-J.	2016	Population genetic diversity and genetic structure of *Spodoptera exigua* around the Bohai Gulf area of China based on mitochondrial DNA signatures	Genetics and Molecular Research	https://doi.org/10.4238/gmr.15039032
46	A (Gen)	Ahmed, I., Huebner, H., Mamoori, Y.I., Buchholz, R.	2017	Identification of newly established *Spodoptera littoralis* cell lines by two DNA barcoding markers	In Vitro Cellular and Developmental Biology - Animal	https://doi.org/10.1007/s11626-016-0118-x
47	A (Gen)	Nagoshi, R.N., Fleischer, S., Meagher, R.L., Hay-Roe, M., Khan, A., Murúa, M.G., Silvie, P., Vergara, C., Westbrook, J.	2017	Fall armyworm migration across the lesser Antilles and the potential for genetic exchanges between North and south American populations	PLoS ONE	https://doi.org/10.1371/journal.pone.0171743
48	A (Gen)	Nagoshi, R.N., Koffi, D., Agboka, K., Tounou, K.A., Banerjee, R., Jurat-Fuentes, J.L., Meagher, R.L.	2017	Comparative molecular analyses of invasive fall armyworm in Togo reveal strong similarities to populations from the eastern United States and the Greater Antilles	PLoS ONE	https://doi.org/10.1371/journal.pone.0181982
49	B (Phe)	Chen, Y., Chen, X., Chen, Y., Wei, H., Lin, S., Tian, H., Lin, T., Zhao, J., Gu, X.	2018	Preparation, characterisation, and controlled release of sex pheromone-loaded MPEG-PCL diblock copolymer micelles for *Spodoptera litura* (Lepidoptera: Noctuidae)	PLoS ONE	https://doi.org/10.1371/journal.pone.0203062
50	B (Phe)	Cruz-Esteban, S.,· Rojas, J.C., Sanchez-Guillen, D., Cruz-Lopez, L.,· Malo, E.A.	2018	Geographic variation in pheromone component ratio and antennal responses, but not in attraction, to sex pheromones among fall armyworm populations infesting corn in Mexico	Journal of Pest Science	https://doi.org/10.1007/s10340-018-0967-z
51	A (Gen)	Donga, T.K., Meadow, R.	2018	Determination of genetic diversity in *Chilo partellus*, *Busseola fusca*, and *Spodoptera frugiperda* infesting sugarcane in southern Malawi using DNA barcodes	Insects	https://doi.org/10.3390/insects9030074
52	A (Gen)	Nagoshi, R.N., Goergen, G., Tounou, K.A., Agboka, K., Koffi, D., Meagher, R.L.	2018	Analysis of strain distribution, migratory potential, and invasion history of fall armyworm populations in northern Sub-Saharan Africa	Scientific Reports	https://doi.org/10.1038/s41598-018-21954-1

53	A (Gen)	Otim, M.H., Tay, W.T., Walsh, T.K., Kanyesigye, D., Adumo, S., Abongosi, J., Ochen, S., Sserumaga, J., Alibu, S., Abalo, G., Asea, G., Agona, A.	2018	Detection of sister-species in invasive populations of the fall armyworm *Spodoptera frugiperda* (Lepidoptera: Noctuidae) from Uganda	PLoS ONE	https://doi.org/10.1371/journal.pone.0194571
54	A (Gen)	Tahir, H.M., Noor, A., Mehmood. S., Sherawat, S.M., Qazi, M.A.	2018	Evaluating the accuracy of morphological identification of insect pests of rice crops using DNA barcoding	Mitochondrial DNA Part B: Resources	https://doi.org/10.1080/23802359.2018.1532334
55	A (Gen)	Assefa, Y.	2019	Molecular identification of the invasive strain of *Spodoptera frugiperda* (JE smith) (Lepidoptera: Noctuidae) in Swaziland	International Journal of Tropical Insect Science	https://doi.org/10.1007/s42690-019-00018-5
56	A (Gen)	Meagher, Jr.R.L.; Koffi, D., Agboka, K., Tounou, A.K,, Agbevohia, K.A., Amouze, T.R., Adjévi, K.M., Nagoshi, R.N.	2019	Comparison of pheromone trap design and lures for *Spodoptera frugiperda* in Togo and genetic characterization of moths caught	Entomologia Experimentalis et Applicata	https://doi.org/10.1111/eea.12795
57	A (Gen)	Nagoshi, R.N., Dhanani, I., Asokan, R., Mahadevaswamy, H.M., Kalleshwaraswamy, C.M., Sharanabasappa, Meagher, R.L.	2019	Genetic characterization of fall armyworm infesting South Africa and India indicate recent introduction from a common source population	PLoS ONE	https://doi.org/10.1371/journal.pone.0217755
58	A (Gen)	Nagoshi, R.N., Goergen, G., Plessis, H.D., Van den Berg, J., Meagher, R.Jr.	2019	Genetic comparisons of fall armyworm populations from 11 countries spanning sub-Saharan Africa provide insights into strain composition and migratory behaviors	Scientific Reports	https://doi.org/10.1038/s41598-019-44744-9
59	A (Gen)	Nagoshi, R.N., Nagoshi, B.Y., Cañarte, E., Navarrete, B., Solórzano, R., Garcés-Carrera, S.	2019	Genetic characterization of fall armyworm (*Spodoptera frugiperda*) in Ecuador and comparisons with regional populations identify likely migratory relationships	PLoS ONE	https://doi.org/10.1371/journal.pone.0222332
60	A (Gen)	Chen, D., Chen, Z., Xu, H., Lin, F.	2020	Host type identification of *Spodoptera frugiperda* invading in Guangzhou by different molecular markers	Journal of South China Agricultural University	https://doi.org/10.7671/j.issn.1001-411X.201906016
61	B (Phe)	Di, X., Liu, J., Wu, C., Yan, B., Yu, X., Yang, M., Morrison, W.	2020	Delayed Mating with Multiple Partners Decreases Indexes of Mating in Female and Male *Spodoptera litura* (Lepidoptera: Noctuidae)	Environmental Entomology	https://doi.org/10.1093/ee/nvaa069
62	A (Gen)	Gilal, A.A., Bashir, L., Faheem, M., Rajput, A., Soomro, J.A., Kunbhar, S., Mirwani, A.S., Tanzeela-ul-Zahra, Mastoi, G.S., Sahito, J.G.M.	2020	First Record of Invasive Fall Armyworm (*Spodoptera frugiperda* (Smith) (Lepidoptera: Noctuidae)) in Corn Fields of Sindh, Pakistan	Pakistan Journal of Agricultural Research	https://doi.org/10.17582/journal.pjar/2020/33.2.247.252
63	C (Gen + Phe)	Haenniger, S., Goergen, G., Akinbuluma, M.D., Kunert, M., Heckel, D.G., Unbehend, M.	2020	Sexual communication of *Spodoptera frugiperda* from West Africa: Adaptation of an invasive species and implications for pest management	Scientific Reports	https://doi.org/10.1038/s41598-020-59708-7

64	A (Gen)	Nagoshi, K.L., Allan, S.A., Meagher, R.L., Showler, A.	2020	Assessing the Use of Wing Morphometrics to Identify Fall Armyworm (Lepidoptera: Noctuidae) Host Strains in Field Collections	Journal of Economic Entomology	https://doi.org/10.1093/jee/toz344
65	A (Gen)	Nanayakkara. D., Jayatilake, D., Kodithuwakku, S.	2020	Development of a single nucleotide polymorphism-based DNA marker for fall armyworm (Lepidoptera: Noctuidae) biotyping: a case study from the fall armyworm outbreak in Sri Lanka	Canadian Entomologist	https://doi.org/10.4039/tce.2020.52
66	A (Gen)	Tsai, C.L., Chu, I.H., Chou, M.H., Chareonviriyaphap, T., Chiang, M.-Y., Lin, P.-A., Lu, K.-H., Yeh, W.-B.	2020	Rapid identification of the invasive fall armyworm *Spodoptera frugiperda* (Lepidoptera, Noctuidae) using species-specific primers in multiplex PCR	Scientific Reports	https://doi.org/10.1038/s41598-020-73786-7
67	A (Gen)	Acharya, R., Akintola, A.A., Malekera, M.J., Kamulegeya, P., Nyakunga, K.B., Mutimbu, M.K., Shrestha, Y.K., Hemayet, J.S.M., Hoat, T.X., Dao, H.T., Park, J.-H., Kim, I., Nam, M., Lee, S.-J., Kim, S.-M., Hwang, H.-S., Lee, K.-Y.	2021	Genetic relationship of fall armyworm (*Spodoptera frugiperda*) populations that invaded Africa and Asia	Insects	https://doi.org/10.3390/insects12050439
68	A (Gen)	Blas, G.S., Baudino, E.M., Dias, F.M.S., Dolibaina, D.R., Specht, A., Casagrande, M.M., Cornejo, P., Giraudo, W.G., Mielke, O.H.H.	2021	Molecular characterization and phylogenetic assessment of agricultural-related noctuids (Lepidoptera: Noctuidae) of South America	Revista Brasileira de Entomologia	https://doi.org/10.1590/1806-9665-RBENT-2021-0104
69	A (Gen)	Cokola, M.C., Ndjadi, S.S., Bisimwa, E.B., Ahoton, L.E., Francis, F.	2021	First report of *Spodoptera frugiperda* (Lepidoptera: Noctuidae) on Onion (Allium cepa L.) in South Kivu, Eastern DR Congo	Revista Brasileira de Entomologia	https://doi.org/10.1590/1806-9665-RBENT-2020-0083
70	A (Gen)	Mahat, K., Mitchell, A., Zangpo, T.	2021	An updated global COI barcode reference data set for Fall Armyworm (*Spodoptera frugiperda*) and first record of this species in Bhutan	Journal of Asia-Pacific Entomology	https://doi.org/10.1016/j.aspen.2020.11.013
71	A (Gen)	Nagoshi, R.N., Koffi, D., Agboka, K., Adjevi, A.K.M., Meagher, R.L., Goergen, G.	2021	The fall armyworm strain associated with most rice, millet, and pasture infestations in the Western Hemisphere is rare or absent in Ghana and Togo	PLoS ONE	https://doi.org/10.1371/journal.pone.0253528
72	A (Gen)	Nelly, N., Hamid, H., Lina, E.C. Yunisman	2021	Distribution and genetic diversity of *Spodoptera frugiperda* j. E. smith (noctuidae: Lepidoptera) on maize in west sumatra, indonesia	Biodiversitas	https://doi.org/10.13057/biodiv/d220507
73	A (Gen)	Piggott, M.P., Tadle, F.P.J., Patel, S., Cardenas, G.K., Thistleton, B.	2021	Corn-strain or rice-strain? Detection of fall armyworm, *Spodoptera frugiperda* (JE Smith) (Lepidoptera: Noctuidae), in northern Australia	International Journal of Tropical Insect Science	https://doi.org/10.1007/s42690-021-00441-7

74	A (Gen)	Sarr, · O.M., Garba, M., Bal, A.B., Hima, K., Ndiaye, M., Fossoud, A., · Clamens, A.-L., Tavoillot, J., Gauthier, N.	2021	Strain composition and genetic diversity of the fall armyworm *Spodoptera frugiperda* (Lepidoptera, Noctuidae): new insights from seven countries in West Africa	International Journal of Tropical Insect Science	https://doi.org/10.1007/s42690-021-00450-6
75	A (Gen)	Schlum, K.A., Lamour, K., de Bortoli, C.P., Banerjee, R., Meagher, R., Pereira, E., Murua, M.G., Sword, G.A., Tessnow, A.E., Viteri, D.D., Ramirez, L.A.M., Akutse, K.S., Schmidt-Jeffris, R., Huang, F., Reisig, D., Emrich, S.J., Jurat-Fuentes, J.L.	2021	Whole genome comparisons reveal panmixia among fall armyworm (*Spodoptera frugiperda*) from diverse locations	BMC Genomics	https://doi.org/10.1186/s12864-021-07492-7
76	A (Gen)	Tessnow, A.E., Gilligan, T.M., Burkness, E., Placidi, De Bortoli, C., Jurat-Fuentes, J.L., Porter, P., Sekula, D., Sword, G.A.	2021	Novel real-time PCR based assays for differentiating fall armyworm strains using four single nucleotide polymorphisms	PeerJ	https://doi.org/10.7717/peerj.12195
77	A (Gen)	Withers, A.J., de Boer, J., Chipabika, G., Zhang, L., Smith, J.A., Jones, C.M., Wilson, K.	2021	Microsatellites reveal that genetic mixing commonly occurs between invasive fall armyworm populations in Africa	Scientific Reports	https://doi.org/10.1038/s41598-021-00298-3
78	A (Gen)	Babu, S.R., Perumal, P., Joshi, S., Manoharan, R., Kalyan, D., Penuballi, S., Kalyan, R.K., Mahla, M.K., Rokadia, P., Singh, B.	2022	Detection of the occurrence of the fall armyworm, *Spodoptera frugiperd*a (J.E. Smith) (Lepidoptera: Noctuidae) strains on maize in Southern Rajasthan India	Journal of Plant Diseases and Protection	https://doi.org/10.1007/s41348-022-00640-5
79	A (Gen)	Chen, P., Sun, S., Guan, G., Haack, R.A., Ye, H., Liu, X.	2022	Distribution characteristics of *Spodoptera frugiperda* (Lepidoptera: Noctuidae) winter populations in typical Mountain agricultural area of Southwest China	International Journal of Pest Management	https://doi.org/10.1080/09670874.2022.2103202
80	A (Gen)	Kamweru, I., Anani, B.Y., Beyene, Y., Makumbi, D., Adetimirin, V.O., Prasanna, B.M., Gowda, M.	2022	Genomic Analysis of Resistance to Fall Armyworm (*Spodoptera frugiperda*) in CIMMYT Maize Lines	Genes	https://doi.org/10.3390/genes13020251
81	A (Gen)	Nagoshi, R.N., Goergen, G., Koffi, D., Agboka, K., Adjevi, A.K.M., Du Plessis, H., Van den Berg, J., Tepa-Yotto, G.T., Winsou, J.K., Meagher, R.L., Brévault, T.	2022	Genetic studies of fall armyworm indicate a new introduction into Africa and identify limits to its migratory behavior	Scientific Reports	https://doi.org/10.1038/s41598-022-05781-z
82	A (Gen)	Park, S.R., Lee, D.E., Nam, H.Y., Kim, J., Lee, S.H., Kim, J.H.	2022	Development of Multiplex PCR-based Protocols for Simultaneous Caterpillar Diagnosis of Three *Spodoptera* and One *Mamestra* Species (Lepidoptera: Noctuidae)	Journal of Economic Entomology	https://doi.org/10.1093/jee/toac076

83	A (Gen)	Sathyan, T., Sathiah, N., Mohankumar, S., Balasubramani, V., Kokiladevi, E., Ravikesavan, R., Srinivasan, T., Kennedy, J.S.	2022	Genetic characterization of fall armyworm (*Spodoptera frugiperda*, J.E. Smith) feeding on pearl millet (*Pennisetum glaucum* L.R.Br.) in Tamil Nadu	Madras Agricultural Journal	https://doi.org/10.29321/MAJ.10.000618
84	A (Gen)	Tay, W.T., Rane, R.V., Padovan, A., Walsh, T.K., Elfekih, S., Downes, S., Nam, K., d'Alençon, E., Zhang, J., Wu, Y., Nègre, N., Kunz, D., Kriticos, D.J., Czepak, C., Otim, M.H., Gordon, K.H.J.	2022	Global population genomic signature of *Spodoptera frugiperda* (fall armyworm) supports complex introduction events across the Old World	Communications Biology	https://doi.org/10.1038/s42003-022-03230-1
85	A (Gen)	Yousaf, S., Rehman, A., Masood, M., Ali, K., Suleman, N.	2022	Occurrence and molecular identification of an invasive rice strain of fall armyworm *Spodoptera frugiperda* (Lepidoptera: Noctuidae) from Sindh, Pakistan, using mitochondrial cytochrome c oxidase I gene sequences	Journal of Plant Diseases and Protection	https://doi.org/10.1007/s41348-021-00548-6
86	A (Gen)	Ramos-Hernández, E., Ortíz-García, C.F., Cordova-Sánchez, S., Castellanos-Potenciano, B.P.	2023	PCR molecular identification of the fall armyworm in the Chontalpa region, Tabasco, Mexico	Agroproductividad	https://doi.org/10.32854/agrop.v16i3.2369
87	A (Gen)	Ishizuka, T.K., Cordeiro, E.M.G., Alves-Pereira, A., de Araujo Batista, C.E., Murua, M.G., Pinheiro, J.B., Sethi, A., Nagoshi, R.N., Foresti, J., Zucchi, M.I.	2023	Population genomics of fall armyworm by genotyping-by-sequencing: Implications for pest management	PLoS ONE	https://doi.org/10.1371/journal.pone.0284587
88	A (Gen)	Malekera, M.J., Mamba, D.M., Bushabu, G.B., Murhula, J.C., Hwang, H.-S., Lee, K.-Y.	2023	Genetic Diversity of the Fall Armyworm *Spodoptera frugiperda* (J.E. Smith) in the Democratic Republic of the Congo	Agronomy	https://doi.org/10.3390/agronomy13082175
89	A (Gen)	Navasero, M.M., Navasero, M.V., Aquino, M.F.G., Navasero, J.M.M., Ginez, V.B., Cabusas, J.V.B., Sapin, G.D., Caoili, B.L.	2023	Morphological, molecular and biological characterization of *Spodoptera frugiperda* (J.E. Smith) from Rice in Northern Luzon, Philippines	International Journal of Agricultural Technology	http://www.ijat-aatsea.com ISSN 2630-0192
90	A (Gen)	Ndung'u, K.E., Khamis, F.M., Ajene, I.J., Mbogo, K.O., Akutse, K.S.	2023	*Spodoptera frugiperda* population structure and influence of farmers' practices on gut biodiversity for sustainable management of the pest in Kenya	Frontiers in Ecology and Evolution	https://doi.org/10.3389/fevo.2023.1235558
91	A (Gen)	Neath, D.O., Robinson, D.E., Tennant, P.F.	2023	Insights into the genetic diversity of *Spodoptera* spp. (Lepidoptera: Noctuidae) affecting vegetable crops in Jamaica	Phytoparasitica	https://doi.org/10.1007/s12600-023-01099-2
92	B (Phe)	Rupali, J.S., Ramya, N., Sagar, D., Padala, V.K., Madhuri, E.V., Subramanian, S.	2023	Reproductive behaviour in different aged adults of fall armyworm, *Spodoptera frugiperda* (J. E. Smith)	Current Science	https://doi.org/10.18520/cs/v125/i3/309-316

93	A (Gen)	Sathyan, T., Sathiah, N., Mohankumar, S., Balasubramani, V., Kokiladevi, E., Ravikesavan, R., Kennedy, J.S.	2023	Molecular characterization of the invasive fall armyworm, *Spodoptera frugiperda* (J.E. Smith) feeding on finger millet, Eleusine coracana (L.) Gaertn	Cereal Research Communications	https://doi.org/10.1007/s42976-022-00338-2
94	A (Gen)	Sinha, T., Narayana, S., Arya, V., Kandan, A., Raju, S.V.S., Samal, I.	2023	Development of a loop-mediated isothermal amplification assay for accurate and rapid identification of *Spodoptera frugiperda* in maize from India	Cereal Research Communications	https://doi.org/10.1007/s42976-023-00462-7
95	B (Phe)	Tabata, J., Nakano, R., Yasui, H., Nakamura, K., Takehara, K., Matsuda, H., Ikenoue, Y., Kusuhata, Y., Kinjo, K., Nakama, K., Tsuha, Y., Ishikawa, Y.	2023	Sex pheromone of the fall armyworm, *Spodoptera frugiperda*: identification of a trace component that enhances attractiveness and specificity	Entomologia Experimentalis et Applicata	https://doi.org/10.1111/eea.13287
96	A (Gen)	Tay, W.T., Kuniata, L., James, W., Walsh, T.	2023	Confirmation of *Spodoptera frugiperda* (Lepidoptera: Noctuidae) in Papua New Guinea by molecular diagnostics of mitochondrial DNA COI gene	BioInvasions Records	https://doi.org/10.3391/bir.2023.12.1.09
97	C (Gen + Phe)	Akinbuluma, M.D., van Schaijk, R.A.H., Roessingh, P., Groot, A.T.	2024	Region-Specific Variation in the Electrophysiological Responses of *Spodoptera frugiperda* (Lepidoptera: Noctuidae) to Synthetic Sex Pheromone Compounds	Journal of Chemical Ecology	https://doi.org/10.1007/s10886-024-01479-w
98	A (Gen)	Cabusas, J.V.B., Latina, R.A., Caoili, B.L.	2024	Genetic diversity and population structure of the fall armyworm, *Spodoptera frugiperda* (J.E. Smith) (Lepidoptera: Noctuidae) in the Philippines	Journal of Applied Entomology	https://doi.org/10.1111/jen.13307
99	A (Gen)	Lebody, K.A.E., Salim, R.G., El-Sayed, G.M., Mahmoud, S.H.	2024	Identification and Genetic Diversity of *Spodoptera frugiperda* J. E. Smith (Lepidoptera: Noctuidae) in Egypt	Agronomy	https://doi.org/10.3390/agronomy14040809
100	A (Gen)	Li, H., Liang, X., Peng, Y., Liu, Z., Zhang, L., Wang, P., Jin, M., Wilson, K., Garvin, M.R. Wu, K., Xiao, Y.	2024	Novel Mito-Nuclear Combinations Facilitate the Global Invasion of a Major Agricultural Crop Pest	Advanced Science	https://doi.org/10.1002/advs.202305353
101	A (Gen)	Liang, X.-Y., Zhang, L., Li, H.-R., Niu, X.-P., Xiao, Y.-T.	2024	Genetic variation in the triosephosphate isomerase gene of the fall armyworm and its distribution across China	Insect Science	https://doi.org/10.1111/1744-7917.13348
102	C (Gen + Phe)	Patil, S., Nayyar, N., Gracy, G., Patil, J., Kesavan, S., Gopalsamy, S., Aravindram, K., Rajagopal, R., Gopal, A., Munikrishnappa, V.K.T., Mahalakshmi, M.L., Mallasamudra, C., Veeregowda, A., Vaddara, L., Pandi, R.K., Sushil, S.N.,. Thiruvengadam, V.	2024	Biological characterization of the predominant strains of fall armyworm in India with regards to biocontrol agents and pheromone	Current Science	https://doi.org/10.18520/cs/v127/i4/475-482
103	B (Phe)	Rojas, J.C., Roblero, E., Malo, E.A.	2024	Assessment of meso-dispensers for mating disruption of fall armyworm in maize	International Journal of Pest Management	https://doi.org/10.1080/09670874.2024.2399770

104	C (Gen + Phe)	Sisay, B., Tamiru, A., Subramanian, S., Weldon, C.W., Khamis, F., Green, K.K., Anderson, P., Torto, B.	2024	Pheromonal variation and mating between two mitotypes of fall armyworm (*Spodoptera frugiperda*) in Africa	Scientific Reports	https://doi.org/10.1038/s41598-024-53053-9
105	A (Gen)	Srivastava, S., Misra, V., Baitha, A., Pandey, H., Sushil, S.N., Mohan, M., Pathak, A.D., Srivastava, S., Singh, D., Mall, A.K.	2024	Genetic Profiling of *Spodoptera litura* (Noctuidae: Lepidoptera) in Indian Sub-Tropical Sugar Beet	Sugar Tech	https://doi.org/10.1007/s12355-024-01458-1
106	A (Gen)	Yudha, I.K.W., Supartha, I.W., Susila, I.W., Sudiarta, P., Wijaya, I.N., Wiradana, P.A.	2024	New occurrence of corn and rice strains of *Spodoptera frugiperda* (Lepidoptera: Noctuidae) in Bali and Lesser Sunda (Indonesia): Genetic diversity, distribution, and damage	Biodiversitas	https://doi.org/10.13057/biodiv/d250505
107	A (Gen)	Zhang, Q., Zhang, Y., Zhang, K., Liu, H., Gou, Y., Li, C., Haq, I.U., Quandahor, P., Liu, C.	2024	Molecular Characterization Analysis and Adaptive Responses of *Spodoptera frugiperda* (Lepidoptera: Noctuidae) to Nutritional and Enzymatic Variabilities in Various Maize Cultivars	Plants	https://doi.org/10.3390/plants13050597

Figures

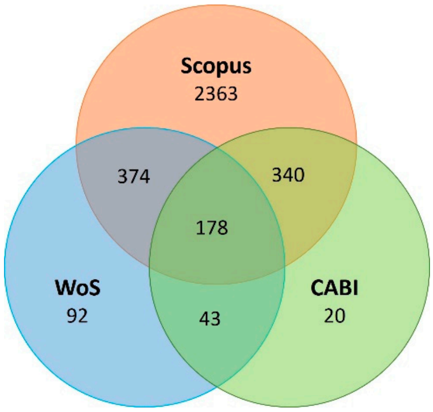

Figure S1. Venn diagram illustrating the number of articles identified specific to each database and the overlap between databases Scopus, Web of Science (WoS), and CABI Digital Library. Numbers indicate the total of articles from each database and the number of articles common to two or in all databases.

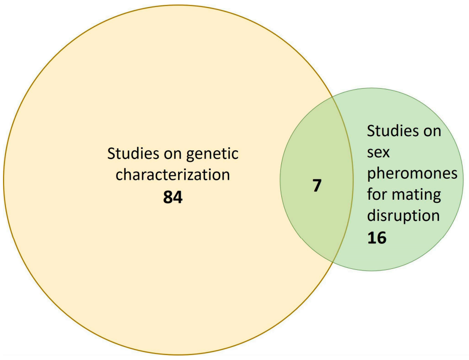

Figure S2. Euler diagram illustrating number of articles per research topic.

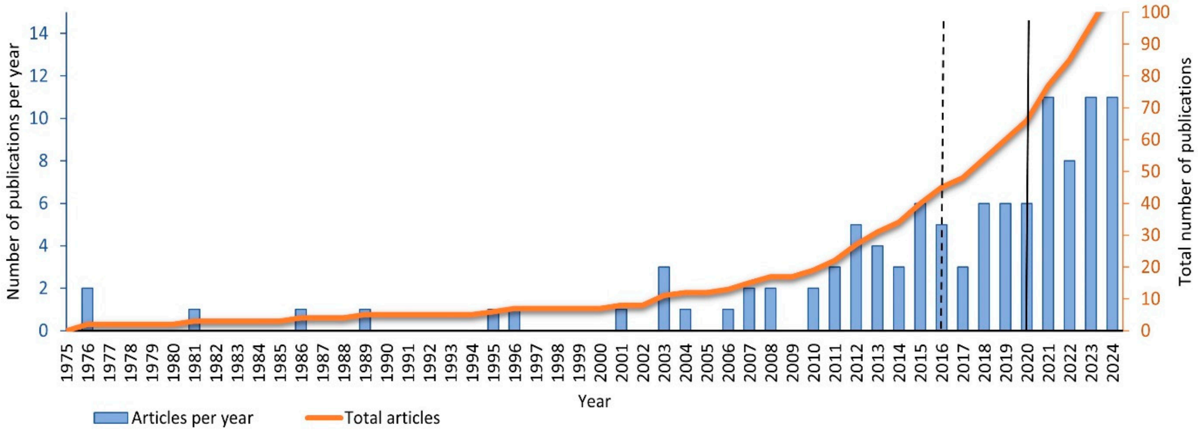

Figure S3. The temporal distribution of articles published from 1975 to 2023. The blue line represents the cumulative total number of publications, while the gray bars represent the number of publications per year. Number of publications over the years, indicating significant increases in publications, particularly after *Spodoptera frugiperda* Smith (Lepidoptera, Noctuidae) in Africa (2016, dashed line) and in Asia (2020, continued line).

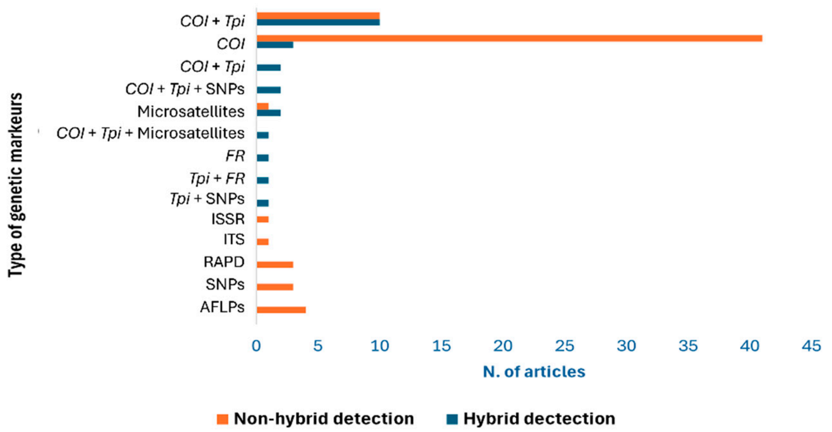

Figure S4. The type of molecular markers used for genetic characterization, and which allowed the detection of hybrids in *Spodoptera frugiperda* (Smith). Each section of the column represents the case of hybrid detection (positive) and non-hybrid detection (negative). Type of genetic markers: *COI* (Cytochrome c oxidase subunit I), *Tpi* (Triosephosphate Isomerase), AFLPs (Amplified Fragment Length Polymorphisms), Microsatellites, SNPs (Single Nucleotide Polymorphisms), RAPD (Random Amplified Polymorphic DNA), ISSR (Inter Simple Sequence Repeats of DNA), ITS (Ribosomal Internal Transcribed Spacer); and FR (for rice).
